# Biocontrol effects of chemical molecules derived from *Beauveria bassiana* against larvae of *Tuta absoluta* (Meyrick) (Lepidoptera: Gelechiidae)

**DOI:** 10.3389/fmicb.2024.1336334

**Published:** 2024-02-12

**Authors:** Perumal Vivekanandhan, Kannan Swathy, Tahani Awad Alahmadi, Mohammad Javed Ansari

**Affiliations:** ^1^Department of General Pathology, Saveetha Dental College and Hospitals, Saveetha Institute of Medical and Technical Sciences (SIMATS), Saveetha University, Chennai, Tamil Nadu, India; ^2^Society for Research and Initiatives for Sustainable Technologies and Institutions, Ahmedabad, Gujarat, India; ^3^Department of Pediatrics, College of Medicine and King Khalid University Hospital, King Saud University, Riyadh, Saudi Arabia; ^4^Department of Botany,.Hindu College Moradabad (Mahatma Jyotiba Phule Rohilkhand University Bareilly), Moradabad, Uttar Pradesh, India

**Keywords:** chemical molecule, acetylcholinesterase, α-carboxylesterase and β-carboxylesterase, larvicide, molecular docking, *Beauveria bassiana*

## Abstract

In this study, we conducted tests on the isolation, identification, characterization, and extraction of chemical molecules from *Beauveria bassiana* against *Tuta absoluta* larvae. The enzyme responses of *T. absoluta* to the crude extract were examined 24 h after treatment, and the number of dead larvae was calculated 24 and 48 h after treatment. Molecular docking studies were conducted to assess the interaction of important molecules with the acetylcholinesterase enzyme. The larvicidal activity of crude chemicals from fungi was high 24 h after treatment, with LC_50_ and LC_90_ values of 25.937 and 33.559 μg/mL, respectively. For a period of 48 h, the LC_50_ and LC_90_ values were 52.254 and 60.450 μg/mL, respectively. The levels of acetylcholinesterase, α-carboxylesterase, and β-carboxylesterase enzymes were lower in the treatment group after 24 h compared to the control group. The GC-MS test revealed that the crude extract consisted mainly of 9,10-octadecadienoic acid, which was the primary compound. Docking results indicated that 9,10-octadecadienoic acid showed a strong interaction with acetylcholinesterase (AChE). Our findings suggest that the chemical molecule 9,10-octadecadienoic acid derived from the entomopathogenic fungus *B. bassiana* is more toxic to *T. absoluta* larvae. We plan to conduct studies to test its effectiveness in semi-field conditions and to evaluate its stability in field conditions. We believe that this 9,10-octadecadienoic acid molecule could be used to control *T. absoluta* larvae in the near future without causing environmental pollution.

## 1 Introduction

*Tuta absoluta* (Meyrick) (Lepidoptera: Gelechiidae), a major tomato pest native to western South America, was first reported in Brazil around 1980 (Souza and Reis, 1992). It has become a significant pest of tomato crops in South America, Europe, Africa, India, and other Asian countries (Tropea Garzia et al., 2012; Zappala et al., 2013). This pest is spreading across borders and causing significant damage to tomato production in both greenhouse and open fields (Fera et al., 2009). *Tuta absoluta* larvae can cause damage to fruits, flowers, stems, and leaves, and also affect the development of crop growth (Biondi et al., 2018). By feeding on the mesophyll and moving in and out of the leaves, a single larva can damage entire plants (Gözel and Kasap, 2015). This insect pest can produce up to 12 generations under favorable climatic conditions, which increases its invasiveness (Biondi et al., 2018). This insect pest can cause yield losses of up to 100% of total production (Desneux et al., 2010).

Chemical control methods have resulted in numerous side effects on human health and the environment, as well as accumulation in soil conditions. When various chemical insecticides, such as permethrin, cypermethrin, monocrotophos, and malathion, are repeatedly used, insects develop resistance to these chemical pesticides (Metcalf, 1989; Gahukar and Kishore, 1995; Vivekanandhan et al., 2021). As a result, various methods of controlling insects using secondary metabolites from plants, bacteria, and fungi have shown greater efficacy (Balumahendhiran et al., 2019; Logeswaran et al., 2019; Pratheeba et al., 2019; Vivekanandhan et al., 2022a,b; Chinnasamy et al., 2023; Kannan et al., 2023; Krutmuang et al., 2023; Perumal et al., 2023a,b,c).

Numerous studies have investigated entomopathogenic fungi, conidia, and their chemical components for controlling *T. absoluta* insect pests, demonstrating their high effectiveness (Zekeya et al., 2019; Bali and Singla, 2022). The entomopathogenic fungi *B. bassiana* and *Metarhizium anisopliae* have shown greater virulence against a wide range of medical and agricultural insect pests (Contreras et al., 2014; Gebremariam et al., 2021; Wang et al., 2021; Bapfubusa Niyibizi et al., 2023; Sharma et al., 2023; Swathy et al., 2023; Vivekanandhan et al., 2023). The cuticle-degrading enzyme chitinase, derived from the entomopathogenic fungi *B. bassiana*, exhibited remarkable insecticidal activity within 5 days against larvae of *B. dorsalis* (Ullah et al., 2023). The conidia of *B. bassiana* and *M. anisopliae* fungi, along with their chemical constituents, have been shown to be effective against *T. absoluta* larvae (Tadele and Emana, 2017; Bali et al., 2023; Giannoulakis et al., 2023). In the current study, chemical molecules were extracted from *B. bassiana*, and their toxic effectiveness was tested on *T. absoluta* larvae in the laboratory. Additionally, molecular docking studies were conducted to investigate the interaction of major molecules with the enzyme acetylcholinesterase.

## 2 Materials and methods

### 2.1 Soil sample

Five soil samples were collected from the rhizosphere areas of healthy cotton plants (*Gossypium herbaceum* L) in Tamil Nadu, India (11.1271° N, 78.6569° E). Soil samples weighing 2.5 kg were collected from depths ranging from 1 to 20 cm and combined, as reported by Vivekanandhan et al. (2020). The soil collected was stored in a sterile bag at 4°C for the subsequent isolation of entomopathogenic fungi.

### 2.2 Insect bait techniques

The soil sample mentioned above was subjected to insect baiting to isolate entomopathogenic fungi, which were subsequently identified using our earlier methodologies and others (Vivekanandhan et al., 2021; Mathulwe et al., 2023). The bait method employing third-instar larvae of *Tenebrio molitor* is an exceptionally sensitive technique used to isolate insect pathogenic fungi from soil. Fifteen *T. molitor* larvae in their third instar were transferred to a plastic container with 300 g of soil, measuring 15 cm (L) × 10 cm (W) × 10 cm (H). After securing the container with a lid, it was placed in an incubator set at 26 ± 1°C and 85% relative humidity. For 15 days, plastic containers were observed twice daily. After collecting the larval cadavers and sterilizing them for 2–3 min with 70% ethanol, the sterile cadavers were transferred to Petri plates (90 mm × 15 mm) containing pre-prepared potato dextrose agar medium PDA (HiMedia, India) (Perumal et al., 2023a). Plates were kept at a relative humidity of 85% and a temperature of 26 ± 2°C for 7 to 10 days. The pure fungal cultures were isolated from the dead larval cadaver according to Balumahendhiran et al. (2019) and Vivekanandhan et al. (2023) and preserved in a biochemical oxygen demand incubator (BOD) (Smartscience, India) at 26 ± 2°C for future experiments.

### 2.3 Morphological confirmation

We first confirmed the morphology of the fungi species by microscopic observations of the fungal colony, pigment production, mycelium structures, and spore shape, following the steps we described in Kumar et al. (2023) and Vivekanandhan et al. (2023). On the clean slide, one drop of lactophenol cotton blue (LCB) stain (HiMedia, India) was mixed with one loop of fungal conidia and thoroughly blended. The prepared glass slides (Borosil, India) were then examined using an Olympus CH20i/India light microscope set to 40× magnification.

### 2.4 Culturing of fungi

The genomic DNA of entomopathogenic fungi was extracted following the method outlined in our previous study (Vivekanandhan et al., 2021; Kumar et al., 2023). A 150-mL conical flask (Borosil, India) containing fungal genomic DNA was prepared with 100 mL of potato dextrose broth (PDB) (HiMedia, Tamil Nadu, India) and autoclaved for 15 min at 120°C. Afterward, the culture media was transferred to a laminar airflow chamber (LAF) with aseptic conditions and cooled, and then 1 × 10^9^ conidia were transferred into the culture medium. As an additional antibacterial measure, 1 ml of chloramphenicol was added during conidial inoculation. The resulting mixture was then incubated at 28 ± 1°C for 5–7 days to promote fungal growth.

### 2.5 Molecular level confirmation

#### 2.5.1 DNA extraction

After being allowed to grow for 5–7 days, the fungal mycelia were filtered through Whatman No. 1 filter paper (HiMedia, India), and genomic DNA was extracted using a fungal mat following the methods described by Kumar et al. (2023). One gram of fungal biomass was lysed in a sterile mortar and pestle with the addition of liquid nitrogen. Once the mycelium was broken up, 2.5 ml of freshly prepared cetyltrimethylammonium bromide (CTAB) lysis buffer was added. The solution was then transferred to clean microtubes. The microtubes were incubated at 60°C for 1 h in a water bath. After incubation, the microtubes were centrifuged at 8,500 rpm for 15 min and then kept at 4°C for 18 min. Following centrifugation, the supernatant was transferred to fresh tubes with a 24:1 ratio of isoamyl alcohol to chloroform. The mixture was gently shaken until an emulsion formed. Twenty minutes later, the microtubes were centrifuged at 13,000 rpm, and the supernatant was transferred to fresh tubes. After mixing 90% ethanol with an equal volume of ice-cold isopropanol, microtubes were incubated at 25°C for 1 h. After collecting the genomic DNA pellet in new tubes, the microtubes were centrifuged at 13,500 rpm for 20 min following the incubation. In conclusion, we used 70% ethanol to purify the genomic DNA. Following the steps outlined by Kumar et al. (2023), the genomic DNA’s purity was checked using 0.8% agarose gel electrophoresis after the ethanol was taken out.

#### 2.5.2 Polymerase chain reaction (PCR)

Forward (GTAGTCATATGCTTGTCTC) and reverse (CTTCCGTCAATTCCTTTAAG) universal primers were utilized to amplify fungal genomic DNA (NS1 and NS2), following the procedures outlined by Kumar et al. (2023). For PCR amplification, a 20 μL reaction volume was used. The reaction mixture contained 1 μl of genomic DNA, 0.2 μl of II DNA polymerase enzyme, 0.1 mg/ml BSA, 3% DMSO, 0.5 M Betaine, and 0.2 mM of each dNTP (dATP, dGTP, dCTP, and dTTP). The PCR procedure consisted of the following stages: annealing at 50°C for 30 s, extension at 72°C for 7 min, and elongation at 72°C for 2 min.

#### 2.5.3 Fungal sequence

The DNA of the entomopathogenic fungus was sequenced at Chromous Biotech Pvt. Ltd, Chennai, India. Sequence outcomes were compared to the GenBank databases using BLAST analysis. The entire species was identified using data obtained from the NCBI GenBank database. Following that, the organisms’ order was synchronized with IN-5 using the CLUSTAL W program. CLUSTALW (BioEdit) (Hall, 1999) is used for aligning multiple sequences. The nucleotide and amino acid sequence homology was determined using MegAlign (DNA Star, Inc., Madison, WI, United States). The fungal sequence was submitted to GenBank (National Centre for Biotechnology Information, NCBI). Phylogenetic analysis was done with MEGA5 software (Tamura et al., 2011), which built a distance matrix and used the neighbor-joining (Saitou and Nei, 1987) methods.

### 2.6 Entomopathogenic fungi *B. bassiana*

Entomopathogenic fungi *B. bassiana* (GenBank: OM346715.1) were sub-cultured on sterilized PDA fungal culture medium with ampicillin (2.7 mg/100 mL) and incubated at 26 ± 2°C for 7–10 days. The liquid broth culture medium for mass culturing of fungi was prepared using our previously established method (Vivekanandhan et al., 2023). Four 500-mL conical flasks were sterilized at 15 psi for 30 min, each containing 250 mL of potato dextrose broth (PDB), composed of 20 g of dextrose, 5 g of peptone, and 1,000 mL of deionized water. The broth media was fortified with 30 mg of ampicillin, an antibacterial agent. Fungal conidia of *B. bassiana* (1 × 10^8^ per/mL) were inoculated and grown in potato dextrose broth (PDB). For 20 days, the flasks were incubated at 26 ± 2°C.

### 2.7 Extraction of secondary metabolites from *B. bassiana*

After 20 days, the entomopathogenic fungi biomass was removed from the broth culture medium using Whatman No. 1 filter paper and washed with distilled water more than five times to remove media particles. Fungal biomass (150 g) was transferred to 1,000 mL glass beakers containing 500 mL of ethyl acetate, which was then mixed with mycelium for 20–25 days at 27 ± 2°C. After the extraction was completed, the liquid portion was separated from the mycelium, and the aqueous phase was filtered using Whatman No. 1 filter paper, following the method described by Balumahendhiran et al. (2019). The secondary metabolites were concentrated in a rotary vacuum evaporator (Superfit-R/150/11, Mumbai, India) at 45–60°C.

### 2.8 Insect collection and maintenance

An egg mass of *T. absoluta* was obtained from the local tomato field (12.106527, 78.136139) according to the methods of de Figueiredo et al. (2023). The egg mass was maintained at a temperature of 28 ± 2°C and a relative humidity of 75–86%, with a 14-h light and 10-h dark photoperiod, as described by de Figueiredo et al. (2023) and Eski et al. (2023). Newly emerged larvae were fed young tomato leaves under laboratory conditions.

### 2.9 Insect larvicidal bioassay

Larval mortality bioassays were conducted following our previous method (Vivekanandhan et al., 2022b). Tomato leaves were immersed in a crude extract derived from *B. bassiana* at various concentrations of 30, 50, 75, 100, and 150 μg/mL. Next, the 4th instar larvae of *T. absoluta* were transferred to the bioassay container containing leaves dipped in the fungal crude extract. Each concentration had three replicates, with each replicate containing 25 larvae. As a negative control, 25 larvae were exposed to 0.05% dimethyl sulfoxide (DMSO). The Abbott (1925) was utilized to calculate and adjust larval mortality percentages after 24 and 48 h of exposure. Probit analysis was utilized to calculate the LC_50_ and LC_90_ values (IBM Corporation, Bengaluru, Karnataka, India).

### 2.10 Larval homogenate preparation

The larval tissue was thoroughly homogenized with 2 mL of PBS buffer and then centrifuged at −4°C for 15 min at a speed of 10,000 rpm. After removing any solid or cellular debris waste, the supernatant was poured into a clean centrifuge tube, placed on ice, and used immediately for acetylcholinesterase, α-carboxylesterase, and β-carboxylesterase enzyme assays as described in our previous study (Vivekanandhan et al., 2022a).

#### 2.10.1 Acetylcholinesterase enzyme assay (AChE)

The control and fungal conidia-treated larvae were placed in separate containers and then cleaned with double-distilled water to remove any excess water using tissue paper. Ellman et al. (1961) utilized acetylcholine iodide as a substrate to assess acetylcholinesterase activity in the larval homogenate. In 850 μL of 100 mM sodium phosphate buffer, 50 μL of larval tissues were mixed at pH 7.5. Each reaction mixture contained 50 μL of 10 mM DTNB and 50 μL of 12.5 mM acetylcholine iodide and was incubated at room temperature for 5 min. The sample’s optical density at 405 nm was measured using a Thermo Scientific Multiskan EX (200–240 V) spectrophotometer with an appropriate blank.

#### 2.10.2 Carboxylesterase enzyme assays

The α- and β-carboxylesterase activities were determined in larval homogenate using the method described by Van Asperen (1962). For the tests, 1 mL of sodium phosphate buffer (pH 7.0, 100 mM) with 250 μM of α- and β-naphthyl acetate was mixed with 30 μL of the homogenate. The solution was then incubated for 30 min at room temperature. Next, 400 μL of 0.3% Fast Blue B in 3.3% sodium dodecyl-sulfate (SDS) was added to each reaction mixture to inhibit the enzymatic process. The mixtures were then left at room temperature for 15 min to allow distinct color to develop. The optical density of the sample was measured for α- and β-carboxylesterase using a Thermo Scientific Multiskan EX-200–240V with a suitable reagent blank. The carboxylesterase activity was measured using a standard curve made with naphthol as the reference standard.

### 2.11 GC-MS analysis

The crude extract of *B. bassiana* was analyzed using gas chromatography-mass spectrometry on an Agilent 6890. GC equipped with 5,973 N mass-selective detectors and an HP-5 capillary column, according to Camele et al. (2023). Helium was used as the carrier gas at a constant flow rate of 1.0 ml/min. The 0.2 μl sample was injected using a 20:1 split while maintaining temperatures of 230°C and 150°C (Camele et al., 2023).

### 2.12 Molecular docking analysis

The protein data (CID: 107568) were used to retrieve the crystal structures, which were then assembled using a protein preparation wizard (Navinraj et al., 2023). The chemical structure that had been isolated was verified for accuracy, and hydrogen atoms were added to neutralize the side chains that are not in close proximity to the binding cavity or involved in forming a salt bridge. The site map module, which utilizes novel analytical and search methods to generate binding site information, predicted the active site of the targeted protein (CID: 107568). During the initial search step of site map prediction, grid points were characterized using the method developed by de Sena Filho et al. (2023). The protein has multiple target binding sites on its surface that can bind the ligand to the receptor. The ligand preparation method was used to generate multiple conformations of the input molecule, including different ionization stages, tautomers, stereochemistries, and molecular conformations, in order to filter out molecules based on various criteria. Finally, the ligand was optimized using the Optimized Potentials for Liquid Simulations (OPLS-2005) force field with the default parameters.

### 2.13 Statistical analysis

The mortality data was analyzed using ANOVA with logarithmic, arcsine, and square root transformations of percentages. The mean of five replications was used to calculate the observed readings. Differences in the data values among the different treatment groups for larvae were evaluated using Tukey’s multiple range test (*p* < 0.05) with Minitab^®^ 17 software. Sigma Plot-12 (Microcal Software) was utilized to calculate the statistical changes for estimating mid-gut enzyme activity. The lethal dosage (LC_50_ and LC_90_) against larvae after 24 and 48 h was estimated using Probit analysis in conjunction with the Minitab^®^17 software package, with a 95% confidence interval.

## 3 Results

### 3.1 Morphology and taxonomy of *Beauveria*

**FIGURE 1:**
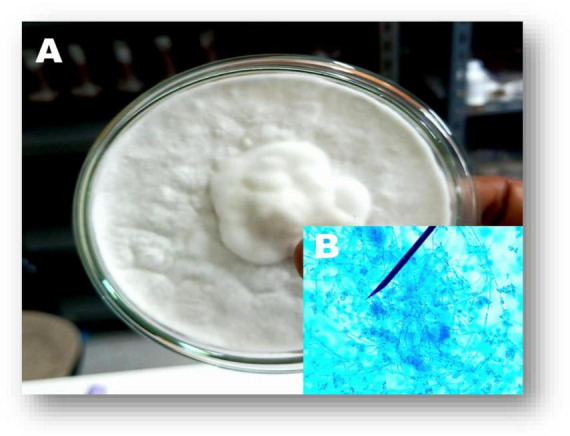
Seven-day-old culture of *B. bassiana* on Potato Dextrose Agar media (GenBank: OM346715.1). **(A)** Fungal culture. **(B)** Fungal conidia.

The morphological features of entomopathogenic fungi (*Beauveria* species) were characterized. The morphological evaluation results revealed oval-shaped conidia and a white colony during microscopic observations (Figures 1A, B). After cultures of *B. bassiana* had completed their normal mycelial and spore development, sterile mycelial growth consistently developed on the surface of the potato dextrose agar media. The new growth appeared on the surface of the culture either as a flat and spreading layer or as a loose, cottony efflorescence (see Figures 1A, B). After nine months, *B. bassiana*, which initially produced white spore masses in culture, yielded sparse masses of pale cream. In this study, it was discovered that mycelium is initially always white. However, as the cultures mature, the color of the mycelium in some of them gradually changes to resemble that of the spores, which typically develop on the surface of the cultures. Table 1 summarizes the morphological characteristics of the listed *B. bassiana*, including conidial shape and size, non-indigenous cell, and colony.

**TABLE 1:** Morphological and microscopic characteristics of *B. bassiana*.

	Colony	Conidia size (μm)	Conidial shape
*Beauveria bassiana*	White and often turning yellowish-white	2.5 to 3.5 μm in length and 1.65 to 2.9 μm in width	Globose, subglobose, or broadly ellipsoid, with ellipsoid being rare.
**Taxonomy information of *Beauveria bassiana***			
Super-kingdom	Eukaryota		
Kingdom	Fungi		
Phylum	Ascomycota		
Class	Sordariomycetes		
Order	Hypocreales		
Family	Cordycipitaceae		
Genus	*Beauveria*		
Species	*Beauveria bassiana*		

### 3.2 Molecular level confirmation

**FIGURE 2:**
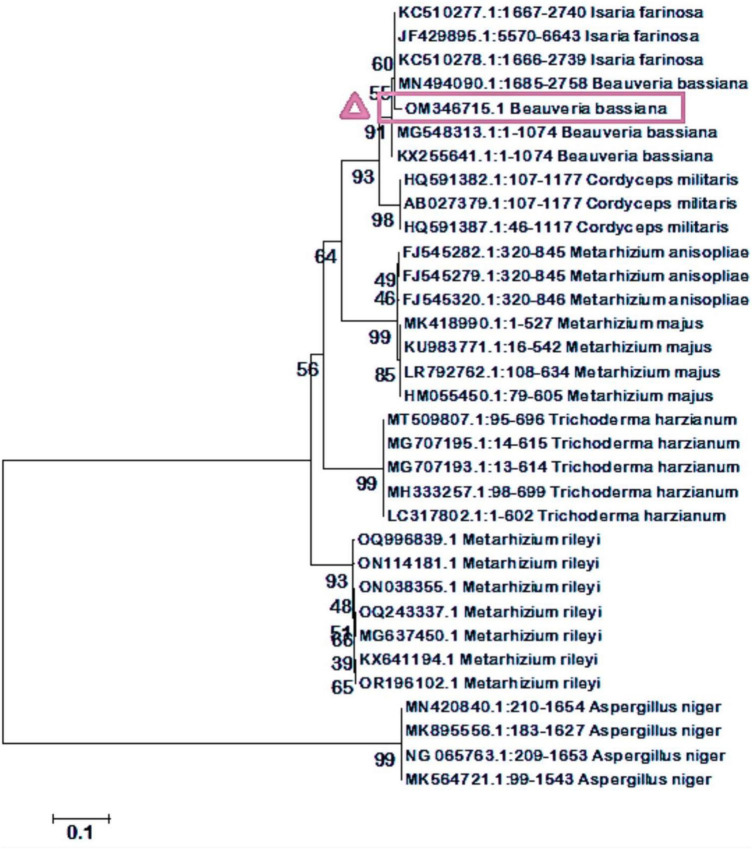
A phylogenetic tree of the stages of evolution of isolated entomopathogenic fungi has been generated using the neighbor-joining tree method. Our isolated fungal strains were identical to *B. bassiana*.

An 18S rDNA gene universal primer was used to amplify fungal genomic DNA. A gel documentation unit was utilized to examine the amplified DNA fragments to confirm their purity. The amplified DNA fragment size range obtained was 426 base pairs (bp). The quality of the amplified DNA molecule sequences was assessed. The fungal DNA sequence was submitted to the NCBI’s GenBank database. The accession number for *Beauveria bassiana* is OM346715.1. The BLAST search for the 18S rRNA sequence revealed a perfect match with previously reported *B. bassiana* cultures. To assess the evolutionary proximity of isolated entomopathogenic fungi, the neighbor-joining tree method was employed (Figure 2).

### 3.3 Larvicidal activity

**FIGURE 3:**
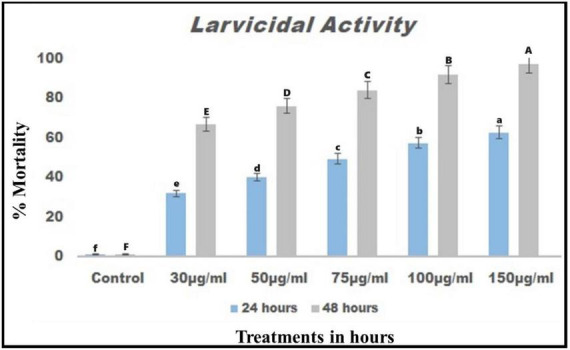
Chemical constituents from *B. bassiana* were tested for larval toxicity against *T. absoluta* larvae after 24 and 48 h of treatment.

The larval toxicity effects of chemical constituents derived from *B. bassiana* resulted in high larvicidal activity against *T. absoluta* larvae 24 h after treatment. It exhibited lower LC_50_ and LC_90_ values of 25.937 μg/mL and 33.559 μg/mL, respectively (Figure 3). After 48 h post-treatment, the LC_50_ and LC_90_ values were 52.254 μg/mL and 60.450 μg/mL, respectively (see Figure 3). Overall, the results suggested that chemical constituents derived from *B. bassiana* exhibit remarkable insect larvicidal activity, causing more than 80% mortality at 24 and 48 h post-treatment.

### 3.4 Molecular docking studies of 9,10-octadecadienoic acid with target proteins

**FIGURE 4:**
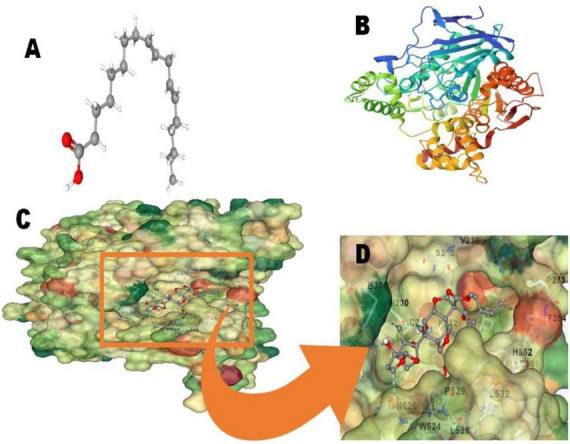
Molecular docking analysis; **(A)** 9 10-octadecadienoic acid molecules (Compound CID: 107568); **(B)** acetylcholinesterase (AChE) protein (PDB ID: 1H22); **(C,D)**: 2D and **(C,D)** Connolly surface views of acetylcholinesterase protein with 9 10-octadecadienoic acid.

In the *B. bassiana* crude extract, 9,10-octadecadienoic acid was identified as a major chemical compound. Therefore, we conducted molecular docking studies with the *T. absoluta* larval acetylcholinesterase (AChE) protein. The 9,10-octadecadienoic acid molecule (Compound CID: 107568) exhibited a high binding affinity with the acetylcholinesterase (AChE) protein (PDB ID: 1H22). The docking score is −7.5, the cavity volume is 1,062 Å3, the docking size (x, y, z) is 23, and the center (x, y, z) is 1, 62, and 67, as illustrated in Figures 4A–D.

### 3.5 Biochemical analysis

**FIGURE 5:**
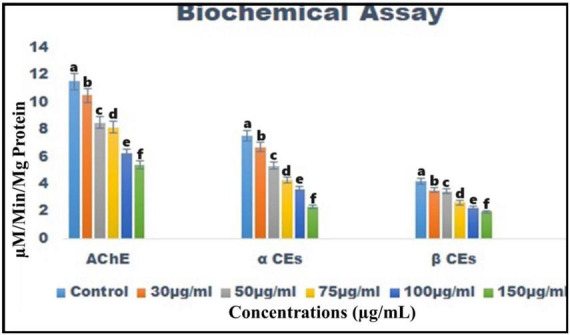
Biochemical analysis of *B. bassiana*-derived chemical constituents on acetylcholinesterase, α-carboxylesterase, and β-carboxylesterase enzymes in the larvae of *T. absoluta*.

Chemical constituents derived from *B. bassiana* reduce the levels of the acetylcholinesterase (AChE) enzyme in *T. absoluta* 4th instar larvae from 11.54 ± 0.3 to 5.41 ± 0.5 M/min/mg larval protein [*F*_(5, 12)_ = 280.360; *p* < 0.01] (Figure 5).

The α-carboxylesterase enzyme levels were similarly reduced [from 7.55 ± 0.5 to 2.33 ± 0.5 M/min/mg larval protein; *F*_(5, 12)_ = 155.850; *P* < 0.01] (Figure 5), and the β-carboxylesterase enzymes were significantly reduced compared to the control [from 4.23 ± 0.5 to 1.98 ± 0.5 M/min/mg larval protein; *F*_(5, 12)_ = 135.263; *p* < 0.01] (Figure 5).

### 3.6 GC-MS analysis

GC-MS analysis revealed six major chemical constituents in the crude extracts of the entomopathogenic fungi *B. bassiana*. The major chemical constituents identified were n-hexadecanoic acid (16.13%), 9,10-octadecadienoic acid (35.47%), 9-eicosyne (13.17%), n-heptacosane (8.36%), tetratetracontane (12.15%), and 7-hexyl eicosane (7.95%) (Table 2). These chemical components may have insect larvicidal activity.

**TABLE 2:** Identification of chemical constituents from entomopathogenic fungi *B. bassiana* derived crude extract using GC-MS analysis.

S. No.	Compound name	Molecular structure	Formula	Area (%)	Biological Activity
1	9-eicosyne	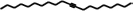	C_20_H_38_	13.17	Anti-microbial activity
2	n-hexadecanoic acid	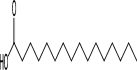	C16H32O2	16.13	Pesticidal activity
3	9,10-octadecadienoic acid	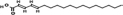	C_18_H_32_O_2_	35.47	Anti-bacterial
4	Heptacosane	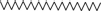	C_27_H_56_	8.36	Anti-microbial activity
5	n-tetratetracontane	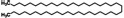	C_34_H_70_	8.36	Antioxidant as well as cytoprotective activities
6	7-hexyl eicosane	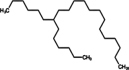	C_26_H_54_	7.95	Antioxidant, and antimicrobial activity

## 4 Discussion

Preliminary identified the isolated entomopathogenic fungi as *Beauveria* species through microscopic observations, such as the presence of white colonies, white pigment production, and oval-shaped conidial structures. We confirmed that the fungal species belong to the *Beauveria* genus based on the microscopic characteristics and taxonomic key (MacLeod, 1954). Similarly, Wargane et al. (2020) reported morphological characteristics that were consistent with our findings. Similar to the present study, Vivekanandhan et al. (2018, 2020) reported that *B. bassiana* was isolated from the soil and exhibited morphological features like conidia shapes and pigment production. The size range of the amplified DNA fragments was 426 base pairs (bp). The neighbor-joining tree method showed 100% identity with the previously published *B. bassiana* when used to determine the evolutionary proximity of isolated entomopathogenic fungi (Figure 2). Similarly, Vivekanandhan et al. (2020) reported that *B. bassiana* was isolated from the soil and exhibited similar fungal morphological features and conidia shapes as the current findings.

Crude extracts obtained from *B. bassiana* have been found to be effective against *T. absoluta* larvae at 24 and 48 h after treatment. The results indicate that crude extracts of *B. bassiana* significantly affect *T. absoluta* larvae 48 h after treatment, resulting in more than 80% mortality (Figure 3). Several previous studies have reported that entomopathogenic fungi and their chemical constituents have shown remarkable toxic efficacy against various developmental stages of *T. absoluta*, including eggs, larvae, pupae, and adults, in both laboratory and field settings (Ndereyimana et al., 2019, 2020; Silva et al., 2020; Hammad et al., 2021; Chouikhi et al., 2022). Similar to the present study, the secondary metabolites from *B. bassiana* were found to be detrimental to *Spodoptera littoralis* on second-instar larvae, resulting in an 86% mortality rate (Abdullah and Sukar, 2021). According to Kona et al. (2014), the ethanolic crude extract from *Azadirachta indica* L. seeds and the petroleum ether crude extract from *Jatropha curcas* L. seeds were highly effective against *T. absoluta* larvae, resulting in up to 100% mortality rates observed 4 days after treatment.

Ghanim and Abdel Ghani (2014) reported using five plant crude extracts against second-instar *T. absoluta* larvae, yielding results similar to those presented in this study. Chinaberry crude extracts had the most significant impact on *T. absoluta* larvae, followed by geranium, onion, and garlic extracts. Hussein et al. (2015) discovered a significant reduction in the *T. absoluta* population after tomato plants were treated with garlic extract. Hussein et al. (2015) found that using essential oil to control *T. absoluta* had a significant impact on fresh weight, shape index, total soluble solids, pericarp thickness, pH, and ascorbic acid levels. Enzymatic analysis revealed that entomopathogenic fungi altered the activity of acetylcholinesterase, α-carboxylesterase, and β-carboxylesterase in *T. absoluta* fourth instar larvae. Additionally, the chemical constituents of *B. bassiana* altered the insect immune system, facilitating infection (Figure 5). In comparison to a control, enzyme levels exhibited dose-dependent activity in response to treatment with a fungal crude extract. Following treatment with the crude extract of the fungus, levels of larval acetylcholinesterase, α-carboxylesterase, and β-carboxylesterase enzymes were significantly reduced. According to the findings, the toxicity of *B. bassiana* crude chemical constituents reduces the levels of acetylcholinesterase, α-carboxylesterase, and β-carboxylesterase enzymes in *T. absoluta* larvae. The enzymes acetylcholinesterase, α-carboxylesterase, and β-carboxylesterase play a crucial role in combating oxidative stress. Similarly, Vivekanandhan et al. (2022a; 2023) found that *M. majus* can decrease enzyme levels in *Spodoptera frugiperda* larvae.

Results of gas chromatography-mass spectrometry (GC-MS) analysis of crude metabolites derived from *B. bassiana* revealed the presence of six major chemical constituents including n-hexadecanoic acid (16.13%), 9,10-octadecadienoic acid (35.47%), 9-eicosyne (13.17%), n-heptacosane (8.36%), tetratetracontane (12.15%), and 7-hexyl eicosane (7.359%) (Table 2). These major chemical components have insecticidal effects on larvae. Previous studies have demonstrated that chemicals extracted from *B. bassiana* have notable insecticidal properties (Vivekanandhan et al., 2018). The primary chemical classes of insecticides derived from plants and microbes primarily act on three target sites within the nervous system: acetylcholinesterase, an enzyme involved in nerve impulse transmission, voltage-gated sodium channels across the nerve membrane, and the acetylcholine receptor. The aforementioned chemical constituents could be involved in targeting these three modes of action. Future research is needed to investigate the mode of action of individual insecticidal molecules, as well as to formulate and test their insecticidal action in the field. In the crude extract of *B. bassiana*, 9,10-octadecadienoic acid was identified as a major chemical compound. Consequently, we conducted molecular docking studies, which revealed a docking score of −7.5, a cavity volume of 1,062 Å3, a docking size of 23 (x, y, z), and a center at 1, 62, and 67 (x, y, z), as depicted in Figures 4C, D. Annapoorani and Manimegalai’s (2013) previous research on insilico molecular docking studies of *Calotropis gigantea*, an aromatic plant with insect repellent properties targeting the OBP of *Culex quinquefasciatus*, supports the findings of our current study. Clarified the information and improved the technical accuracy by using appropriate terminology and providing a more coherent and precise description of the research findings.

Similarly, the insecticidal molecule beta-amyrin successfully docked with OBP. *In silico* molecular docking of insect repellent compounds from *Hyptis suaveolens* yielded similar results (Gaddaguti et al., 2012). According to other research, sitosterol extracted from *H. suaveolens* has a higher binding affinity than the main known odorant-binding protein molecules in decanol. As a result, 9,10-octadecadienoic acid exhibited strong binding and interaction with the insect odorant-binding protein and acetylcholinesterase. Carbamate resistance is present in insect populations, and organophosphates are insensitive to acetylcholinesterase (AChE) and odorant-binding proteins (OBPs), which confer resistance (Annapoorani and Manimegalai, 2013).

## 5 Conclusion

The current research emphasizes the potential of chemical components from the insect-killing fungus *B. bassiana* as efficient and eco-friendly alternatives to chemical insect pest control. Morphological and genetic analysis confirmed the identification of the insect-killing fungus as *B. bassiana*. The toxicity of a raw extract from *B. bassiana* against *T. absoluta* larvae confirms its ability to control insect pest larvae in crops. Results from molecular docking analysis indicate that the primary molecule (9,10-octadecadienoic acid) from the insect-killing fungus *B. bassiana* strongly interacts with the acetylcholinesterase (AChE) protein (PDB ID: 1H22) target proteins, making it effective against *T. absoluta* larvae. These findings open the door for the development of a new larvicidal agent from the insect-killing fungus *B. bassiana*. Further research is planned to isolate the active molecule from the *B. bassiana* raw extract, test it under laboratory and field conditions, evaluate its stability, and develop novel insecticides from insect-killing fungi to combat insect pests.

## Data availability statement

The datasets presented in this study can be found in online repositories. The names of the repository/repositories and accession number(s) can be found in this article. Further enquiries may be directed to the corresponding author.

## Ethics statement

This study presents research on animals that do not require ethical approval for their study.

## Author contributions

PV: Conceptualization, Data curation, Investigation, Methodology, Supervision, Formal analysis, Validation, Visualization, Writing—original draft, Writing—review and editing. KS: Conceptualization, Data curation, Methodology, Resources, Formal analysis, Validation, Visualization, Writing—original draft, Writing—review and editing. TA: Formal analysis, Validation, Visualization, Writing—original draft, Writing—review and editing. MA: Formal analysis, Validation, Visualization, Writing—original draft, Writing—review and editing.

## References

[B1] Abbott W. S. (1925). A method of computing the effectiveness of an insecticide. *J. Econ. Entomol.* 18 265–267. 10.1093/jee/18.2.265a

[B2] Abdullah R. R. Sukar N. A. (2021). Enhancing the efficacy of the biopesticide *Beauveria bassiana* by adding chitosan to its secondary metabolites. *Int. J. Entomol. Res.* 6 30–35.

[B3] Annapoorani C. Manimegalai K. (2013). Screening of medicinal plant *Momordica charantia* leaf for secondary metabolites. *Int. J. Pharm. Res. Dev.* 5 1–6.

[B4] Bali G. K. Maurya D. K. Singh S. K. Pandit R. S. (2023). Morphology, phylogeny, and pathogenicity of *Simplicillium obclavatum* (Hypocreales: Cordycipitaceae) against tomato leafminer, Tuta absoluta (Meyrick) (Lepidoptera: Gelechiidae). *Int. J. Trop. Insect Sci.* 43 355–361. 10.1007/s42690-023-00944-5

[B5] Bali N. Singla A. (2022). Emerging trends in machine learning to predict crop yield and study its influential factors: A survey. *Arch. Comput. Methods Eng.* 29 95–112. 10.1007/s11831-021-09569-8

[B6] Balumahendhiran K. Vivekanandhan P. Shivakumar M. S. (2019). Mosquito control potential of secondary metabolites isolated from *Aspergillus flavus* and *Aspergillus fumigatus*. *Biocatal. Agric. Biotechnol.* 21:101334. 10.1016/j.bcab.2019.101334

[B7] Bapfubusa Niyibizi I. A. Hanna R. Kekeunou S. Membang G. Fiaboe K. K. M. Mahot H. C. (2023). Potential of Cameroon-indigenous isolates of the entomopathogenic fungi *Beauveria bassiana* and *Metarhizium anisopliae* as microbial control agents of the flea beetle *Nisotra uniformis*. *Biocontrol Sci. Technol.* 33 226–240. 10.1080/09583157.2023.2175784

[B8] Biondi A. Guedes R. N. C. Wan F. H. Desneux N. (2018). Ecology, worldwide spread, and management of the invasive South American tomato pinworm, *Tuta absoluta*: Past, present, and future. *Annu. Rev. Entomol.* 63 239–258. 10.1146/annurev-ento-031616-034933 28977774

[B9] Camele I. Sadeek S. A. Racioppi R. Elshafie H. S. (2023). Antimicrobial activity of diffusible and volatile metabolites emitted by *Beauveria bassiana*: Chemical profile of volatile organic compounds (VOCs) Using SPME-GC/MS analysis. *Plants* 12:2854. 10.3390/plants12152854 37571008 PMC10421005

[B10] Chinnasamy R. Govindasamy B. Venkatesh M. Magudeeswaran S. Dhanarajan A. Devarajan N. (2023). Bio-efficacy of insecticidal molecule emodin against dengue, filariasis, and malaria vectors. *Environ. Sci. Pollut. Res.* 30 61842–61862. 10.1007/s11356-023-26290-0 36934179

[B11] Chouikhi S. Assadi B. H. Lebdi K. Belkadhi M. S. (2022). Efficacy of the entomopathogenic fungi *Beauveria bassiana* and *Lecanicillium muscarium* in the control of the tomato leaf miner, *Tuta absoluta* (Meyrick) (Lepidoptera: Gelechiidae). *Egypt. J. Biol. Pest Control* 32 1–8. 10.1186/s41938-022-00640-5

[B12] Contreras J. Mendoza J. E. Martínez-Aguirre M. R. García-Vidal L. Izquierdo J. Bielza P. (2014). Efficacy of enthomopathogenic fungus *Metarhizium anisopliae* against *Tuta absoluta* (Lepidoptera: Gelechiidae). *J. Econ. Entomol.* 107 121–124. 10.1603/EC13404 24665693

[B13] de Figueiredo K. G. de Paiva Silva G. T. Passos L. C. Alves D. S. Biondi A. Carvalho G. A. (2023). Toxicity of *Cinnamomum* spp. essential oil to *Tuta absoluta* and to predatory mirid. *J. Pest Sci.* 1–17. 10.1007/s10340-023-01719-0

[B14] de Sena Filho J. G. de Almeida A. S. Pinto-Zevallos D. Barreto I. C. de Holanda Cavalcanti S. C. Nunes R. (2023). From plant scent defense to biopesticide discovery: Evaluation of toxicity and acetylcholinesterase docking properties for *Lamiaceae monoterpenes*. *Crop Protect.* 164:106126. 10.1016/j.cropro.2022.106126

[B15] Desneux N. Wajnberg E. Wyckhuys K. A. Burgio G. Arpaia S. Narváez-Vasquez C. A. (2010). Biological invasion of European tomato crops by *Tuta absoluta*: Ecology, geographic expansion and prospects for biological control. *J. Pest Sci.* 83 197–215. 10.1007/s10340-010-0321-6

[B16] Ellman G. L. Courtney K. D. Andres V. Featherstone R. M. (1961). A new and rapid colorimetric determination of acetylcholinesterase activity. *Biochem. Pharmacol*. 7, 88–95. 13726518 10.1016/0006-2952(61)90145-9

[B17] Eski A. Erdoğan P. Demirbağ Z. Demir I. (2023). Isolation and identification of bacteria from the invasive pest *Tuta absoluta* (Meyrick) (Lepidoptera: Gelechiidae) and evaluation of their biocontrol potential. *Int. Microbiol.* 1–13. 10.1007/s10123-023-00418-1 37597112

[B18] Fera T. Bluml B. M. Ellis W. M. (2009). Diabetes Ten City Challenge: Final economic and clinical results. *J. Am. Pharm. Assoc*. 49, 383–391. 10.1331/JAPhA.2009.09015 19357068

[B19] Gaddaguti V. Mounika S. Sowjanya K. Rao T. Chakravarthy M. Allu R. (2012). GC-MS analysis and in silico molecular docking studies of mosquito repellent compounds from *Hyptis suaveolens*. *Int. J. Biomass* 1 36–41.

[B20] Gahukar R. T. Kishore P. (1995). Innovative strategy involving judicious pesticide management to control pests of sorghum in India. *J. Entomol. Res.* 19 301–312.

[B21] Gebremariam A. Chekol Y. Assefa F. (2021). Phenotypic, molecular, and virulence characterization of entomopathogenic fungi, *Beauveria bassiana* (Balsam) *Vuillemin*, and *Metarhizium anisopliae* (Metschn.) Sorokin from soil samples of Ethiopia for the development of mycoinsecticide. *Heliyon* 7:e07091. 10.1016/j.heliyon.2021.e07091 34095584 PMC8166759

[B22] Ghanim N. M. Abdel Ghani S. B. (2014). Controlling *Tuta absoluta* (Lepidoptera: Gelechiidae) and *Aphis gossypii* (Hemiptera: Aphididae) by aqueous plant extracts. *Life Sci. J.* 11 299–307.

[B23] Giannoulakis E. Mantzoukas S. Lagogiannis I. Dervisoglou S. Perdikis D. (2023). Efficacy of endophytic wild strains of entomopathogenic fungi against the tomato leafminer *Tuta absoluta* Meyrick) (Lepidoptera: Gelechiidae) in tomato plants. *Egypt. J. Biol. Pest Control* 33 1–9.

[B24] Gözel Ç Kasap I. (2015). Efficacy of entomopathogenic nematodes against the Tomato leafminer, *Tuta absoluta* (Meyrick) (Lepidoptera: Gelechiidae) in tomato field. *Turk. J. Entomol.* 39 229–237.

[B25] Hall T. A. (1999). BioEdit: A user-friendly biological sequence alignment editor and analysis program for Windows 95/98/NT. *Nucleic Acids Symp. Ser*. 41, 95–98.

[B26] Hammad A. M. A. Bashir H. A. A. A. Abdelbagi A. O. Ishag A. E. S. A. Ali M. M. Y. Bashir M. O. (2021). Efficacy of indigenous entomopathogenic fungi for the control of the tomato leafminer *Tuta absoluta* (Meyrick) in Sudan. *Int. J. Trop. Insect. Sci.* 42 1449–1459.

[B27] Hussein N. M. Hussein M. I. Gadel Hak S. H. Shaalan H. S. Hammad M. A. (2015). Effect of two plant extracts and four aromatic oils on *Tuta absoluta* population and productivity of tomato cultivar gold stone. *J. Plant Prot. Pathol.* 6 969–985.

[B28] Kannan S. Perumal V. Yuvaraj A. Pittarate S. Kim J. S. Krutmuang P. (2023). Biodegradation of pesticide in agricultural soil employing entomopathogenic fungi: Current state of the art and future perspectives. *Heliyon* 10:e23406. 10.1016/j.heliyon.2023.e23406 PMC10770572 38187317

[B29] Kona N. E. M. Taha A. K. Mahmoud M. E. (2014). Effects of botanical extracts of Neem (*Azadirachta indica*) and jatropha (*Jatropha curcus*) on eggs and larvae of tomato leaf miner, *Tuta absoluta* (Meyrick) (Lepidoptera: Gelechiidae). *Persian Gulf Crop Prot.* 3 41–46.

[B30] Krutmuang P. Rajula J. Pittarate S. Chanbang Y. Perumal V. Alford L. (2023). Biocontrol efficacy of *Beauveria bassiana* in combination with tobacco short stem and modified lure traps. *Int. J. Trop. Insect Sci.* 43 1591–1600. 10.1007/s42690-023-01063-x

[B31] Kumar C. S. Jacob T. K. Devasahayam S. Rajeshkumar K. C. Lad S. S. D’Silva S. (2023). Metarhizium indicum, a new species of entomopathogenic fungus infecting leafhopper, *Busoniomimus manjunathi* from India. *J. Invert. Pathol.* 198:107919. 10.1016/j.jip.2023.107919 37004918

[B32] Logeswaran C. Vivekanandhan P. Shivakumar M. S. (2019). Chemical constituents of thermal stress induced *Ganoderma applantum* (Per.) secondary metabolites on larvae of *Anopheles stephensi*, *Aedes aegypti* and *Culex quinquefasciatus* and histopathological effects in mosquito larvae. *Biocatal. Agric. Biotechnol.* 20:101253.

[B33] MacLeod D. M. (1954). Investigations on the genera *Beauveria vuill*, and *Tritirachium limber*. *Can. J. Bot.* 32 818–890.

[B34] Mathulwe L. L. Malan A. P. Stokwe N. F. (2023). The occurrence of entomopathogenic fungi in apple orchards and their biocontrol potential against *Eriosoma lanigerum*. *Afr. Entomol.* 31 1–9.

[B35] Metcalf R. L. (1989). Insect resistance to insecticides. *Pest. Sci.* 26 333–358.

[B36] Navinraj S. Boopathi N. M. Balasubramani V. Nakkeeran S. Raghu R. Gnanam R. (2023). Molecular docking of nimbolide extracted from leaves of *Azadirachta indica* L. with protein targets to confirm the antifungal, antibacterial and insecticidal activity. *Indian J. Microbiol.* 63 494–512. 38031617 10.1007/s12088-023-01104-6 PMC10682360

[B37] Ndereyimana A. Nyalala S. Murerwa P. Gaidashova S. (2019). Pathogenicity of some commercial formulations of entomopathogenic fungi on the tomato leaf miner, *Tuta absoluta* (Meyrick) (Lepidoptera: Gelechiidae). *Egypt. J. Biol. Pest Control* 29:70.

[B38] Ndereyimana A. Nyalala S. Murerwa P. Gaidashova S. (2020). Field efficacy of entomopathogens and plant extracts on *Tuta absoluta* Meyrick (Lepidoptera: Gelechiidae) infesting tomato in Rwanda. *Crop Prot.* 134:105183.

[B39] Perumal V. Kannan S. Alford L. Pittarate S. Geedi R. Elangovan D. (2023a). First report on the enzymatic and immune response of *Metarhizium majus* bag formulated conidia against *Spodoptera frugiperda*: An ecofriendly microbial insecticide. *Front. Microbiol.* 14:1104079. 10.3389/fmicb.2023.1104079 36937255 PMC10019823

[B40] Perumal V. Kannan S. Alford L. Pittarate S. Mekchay S. Reddy G. V. (2023b). Biocontrol effect of entomopathogenic fungi *Metarhizium anisopliae* ethyl acetate-derived chemical molecules: An eco-friendly anti-malarial drug and insecticide. *Arch. Insect Biochem. Physiol.* 114, 1–19. 10.1002/arch.22037 37497800

[B41] Perumal V. Kannan S. Pittarate S. Chinnasamy R. Krutmuang P. (2023c). Essential oils from *Acacia nilotica* (Fabales: Fabaceae) seeds: May have insecticidal effects? *Heliyon* 9:e14808. 10.1016/j.heliyon.2023.e14808 PMC10119573 37089397

[B42] Pratheeba T. Vivekanandhan P. Faeza A. N. Natarajan D. (2019). Chemical constituents and larvicidal efficacy of *Naringi crenulata* (Rutaceae) plant extracts and bioassay guided fractions against *Culex quinquefasciatus* mosquito (Diptera: Culicidae). *Biocatal. Agric. Biotechnol.* 19:101137.

[B43] Saitou N. Nei M. (1987). The neighbor-joining method: A new method for reconstructing phylogenetic trees. *Mol. Biol. Evol.* 4, 406–425. 3447015 10.1093/oxfordjournals.molbev.a040454

[B44] Sharma A. Sharma S. Yadav P. K. (2023). Entomopathogenic fungi and their relevance in sustainable agriculture: A review. *Cogent Food Agric.* 9:2180857. 10.1080/23311932.2023.2180857

[B45] Silva A. C. L. Silva G. A. Abib P. H. N. Carolino A. T. Samuels R. I. (2020). Endophytic colonization of tomato plants by the entomopathogenic fungus *Beauveria bassiana* for controlling the South American tomato pinworm, *Tuta absoluta*. *CABI Agric. Biosci.* 1 1–9. 10.1186/s43170-020-00002-x

[B46] Souza J. C. Reis P. R. (1992). *Traça a-do-tomateiro: histórico, reconhecimento, biologia, prejuízos e controle*. Belo Horizonte: EPAMIG.

[B47] Swathy K. Parmar M. K. Vivekanandhan P. (2023). Biocontrol efficacy of entomopathogenic fungi *Beauveria bassiana* conidia against agricultural insect pests. *Environ. Qual. Manag.* 1–11. 10.1002/tqem.22174

[B48] Tadele S. Emana G. (2017). Entomopathogenic effect of *Beauveria bassiana* (Bals.) and *Metarrhizium anisopliae* (Metschn.) on *Tuta absoluta* (Meyrick) (Lepidoptera: Gelechiidae) larvae under laboratory and glasshouse conditions in Ethiopia. *J. Plant Pathol. Microbiol.* 8 411–414.

[B49] Tamura K. Peterson D. Peterson N. Stecher G. Nei M. Kumar S. (2011). MEGA5: Molecular evolutionary genetics analysis using maximum likelihood, evolutionary distance, and maximum parsimony methods. *Mol. Biol. Evol.* 28, 2731–2739. 21546353 10.1093/molbev/msr121 PMC3203626

[B50] Tropea Garzia G. Siscaro G. Biondi A. Zappalà L. (2012). Tuta absoluta, a South American pest of tomato now in the EPPO region: Biology, distribution and damage. *EPPO Bull*. 42, 205–210.

[B51] Ullah S. Naeem H. Murtaza A. Sharif U. Sarfaraz S. Ali F. (2023). Extraction and characterization of cuticle degrading enzymes of *Beauveria bassiana* for enhanced pathogenicity against *Bactrocera dorsalis*. *Plant Prot.* 7 225–235.

[B52] Van Asperen K. (1962). A study of housefly esterases by means of a sensitive colorimetric method. *J. Insect Physiol*. 8, 401–416.

[B53] Vivekanandhan P. Bedini S. Shivakumar M. S. (2020). Isolation and identification of entomopathogenic fungus from Eastern Ghats of South Indian forest soil and their efficacy as biopesticide for mosquito control. *Parasitol. Int.* 76:102099. 10.1016/j.parint.2020.102099 32169659

[B54] Vivekanandhan P. Kavitha T. Karthi S. Senthil-Nathan S. Shivakumar M. S. (2018). Toxicity of Beauveria bassiana-28 mycelial extracts on larvae of *Culex quinquefasciatus* mosquito (Diptera: Culicidae). *Int. J. Environ. Res. Public Health* 15:440. 10.3390/ijerph15030440 PMC5876985 29510502

[B55] Vivekanandhan P. Swathy K. Alford L. Pittarate S. Krutmuang P. (2023). Entomopathogenic fungi based microbial insecticides and their physiological and biochemical effects on *Spodoptera frugiperda* (JE Smith). *Front. Cell. Infect. Microbiol.* 13:1254475. 10.3389/fcimb.2023.1254475 38149005 PMC10750404

[B56] Vivekanandhan P. Swathy K. Alford L. Pittarate S. Subala S. P. R. R. Mekchay S. (2022a). Toxicity of *Metarhizium flavoviride* conidia virulence against *Spodoptera litu*ra (Lepidoptera: Noctuidae) and its impact on physiological and biochemical activities. *Sci. Rep.* 12:16775. 10.1038/s41598-022-20426-x PMC9537412 36202839

[B57] Vivekanandhan P. Swathy K. Shivakumar M. S. (2022b). Identification of insecticidal molecule aucubin from *Metarhizium anisopliae* ethyl acetate crude extract against disease mosquito vector. *Int. J. Trop. Insect Sci.* 42 3303–3318. 10.1007/s42690-022-00828-0

[B58] Vivekanandhan P. Thendralmanikandan A. Kweka E. J. Mahande A. M. (2021). Resistance to temephos in *Anopheles stephensi* larvae is associated with increased cytochrome P450 and α-esterase genes overexpression. *Int. J. Trop. Insect Sci.* 41 2543–2548. 10.1007/s42690-021-00434-6

[B59] Wang H. Peng H. Li W. Cheng P. Gong M. (2021). The toxins of *Beauveria bassiana* and the strategies to improve their virulence to insects. *Front. Microbiol.* 12:705343. 10.3389/fmicb.2021.705343 34512581 PMC8430825

[B60] Wargane V. S. Parate S. R. Bramhankar S. B. Rakhonde P. N. Sonune B. D. Mane K. K. (2020). Cultural and morphological characterizations of *Beauveria bassiana*. *J. Pharm. Phytochem.* 9 591–594.

[B61] Zappala L. Biondi A. Alma A. Al-Jboory I. J. Arno J. Bayram A. (2013). Natural enemies of the South American moth, *Tuta absoluta*, in Europe, North Africa and Middle East, and their potential use in pest control strategies. *J. Pest Sci.* 86, 635–647.

[B62] Zekeya N. Mtambo M. Ramasamy S. Chacha M. Ndakidemi P. A. Mbega E. R. (2019). First record of an entomopathogenic fungus of tomato leafminer, *Tuta absoluta* (Meyrick) in Tanzania. *Biocontrol Sci. Technol.* 29 626–637. 10.1080/09583157.2019.1573972

